# Unusual Ovarian Vein Thrombosis Associated with Urinary Tract Infection: A Case Report

**DOI:** 10.5334/jbsr.2810

**Published:** 2022-04-27

**Authors:** Beom Kyun Pak, In O Sun, Dong Min Kang

**Affiliations:** 1 Presbyterian Medical Center, KR

**Keywords:** multidetector computed tomography, urinary tract infections, urogenital disease, venous thrombosis, thrombophlebitis

## Abstract

**Teaching Point:** Radiologists need to be familiar with that ovarian vein thrombosis can occur as a complication of urinary tract infection.

Ovarian vein thrombosis is a rare disease in which a majority of cases occur during the postpartum period. There are few case reports for ovarian vein thrombosis associated with urinary tract infection in non-postpartum women. We report a case of ovarian vein thrombosis incidentally diagnosed on computed tomography in a patient with symptoms of urinary tract infection.

## Introduction

Ovarian vein thrombosis (OVT) is a rare condition in which a majority of cases occur during the postpartum period; this is called postpartum OVT (POVT). Although it is known to be rare, OVT also occurs in non-postpartum circumstances, such as pelvic inflammatory diseases (PID), pelvic tumors, pelvic surgery, and sepsis, and can occur occasionally without an underlying cause [1]. In the literature, there are few case reports for OVT associated with urinary tract infection (UTI) [2]. Herein, we report a case of UTI-induced OVT.

## Case Report

**Figure 1:**
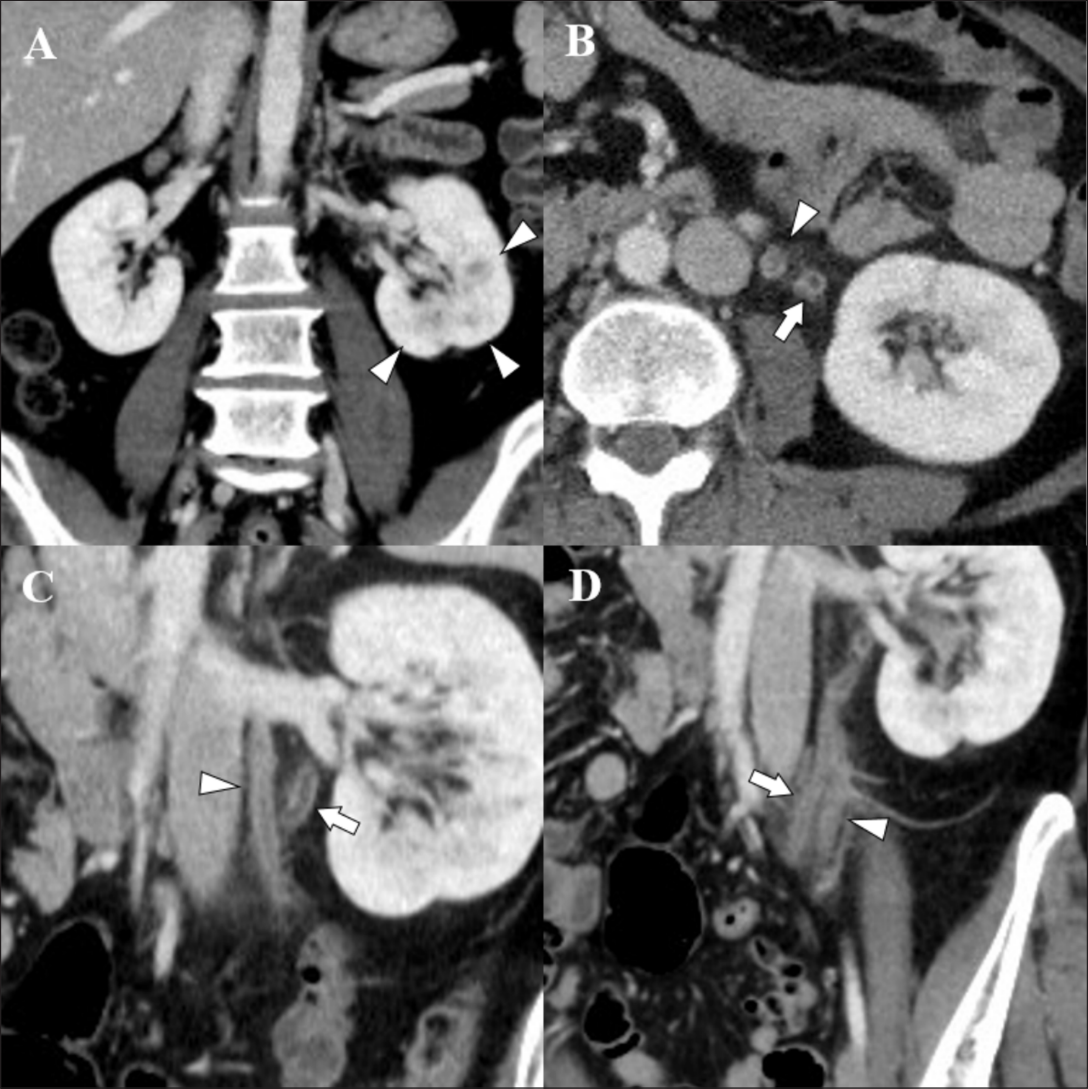
Pre-treatment CT images of a 46-year-old woman with UTI-induced ovarian vein thrombosis. A coronal image (**A**) shows multifocal striated nephrogram (arrowhead) of the left kidney. An axial image (**B**) and oblique coronal images (**C, D**) show enhancing wall thickening of the left ureter (arrow) and hypoattenuated filling defect with enhancing wall of the left ovarian vein (arrowhead). Also, inflammatory fat infiltration around the ureter and ovarian vein is observed.

**Figure 2:**
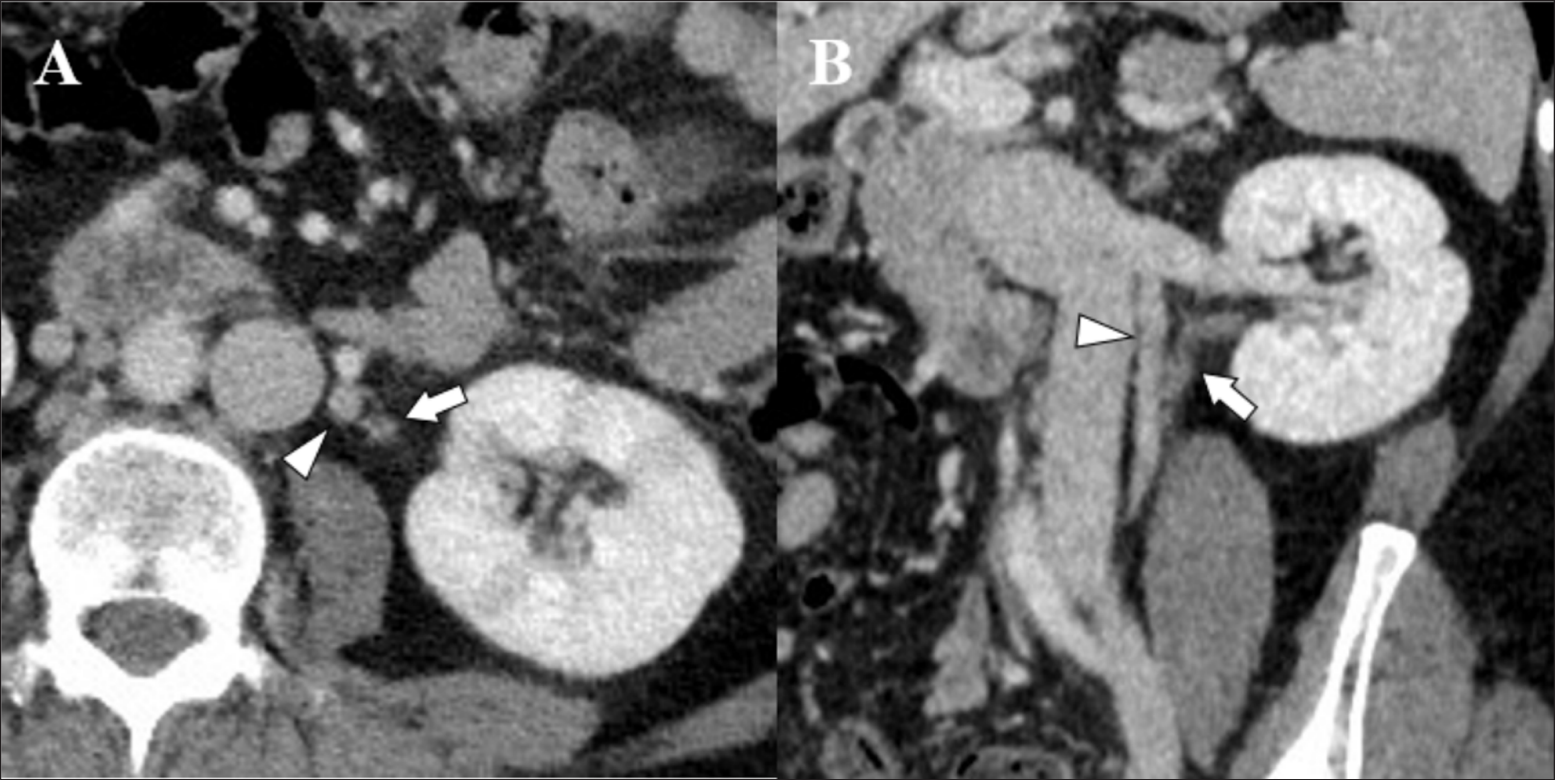
Follow-up CT images taken after 30 days. An axial image (**A**) and an oblique coronal image (**B**) show normalization of the left ureter (arrow) and recanalization of the left ovarian vein (arrowhead).

A 46-year-old female patient visited the emergency department with dysuria and fever. She had a medical history of cystitis. In laboratory tests, pyuria, leukocytosis, and elevated serum C-reactive protein were noted. Urine culture revealed E. coli colonization, but there was no growth of bacteria from the blood culture. CT scan showed multifocal striated nephrogram of the left kidney (***Figure 1A***), suggesting acute pyelonephritis. Additionally, CT findings demonstrated diffuse enhancing wall thickening of the left ureter and urinary bladder, which were considered ureteritis and cystitis. A round, hypoattenuated filling defect with peripheral enhancing wall of the left ovarian vein and perivenous inflammatory fat infiltration were also observed (***Figure 1B–D***). Based on the CT findings, it was presumed to be thrombophlebitis of the left ovarian vein. There was no evidence of PID, such as pelvic fat stranding, ascites, and uterosacral ligament thickening. Upon gynecologic exam, there was no evidence of PID. As a result, she was diagnosed with UTI and complicated septic thrombophlebitis of the left ovarian vein. Fortunately, pulmonary septic embolism was not observed. She was treated with antibiotics and conservative management for 11 days and then discharged. Follow-up CT was taken after 30 days and recanalization of the left ovarian vein was noted (***Figures 2A, B***).

## Discussion

The pathophysiology of POVT is closely related to Virchow’s triad, which describes three factors, hypercoagulability, hemodynamic changes, and endothelial injury. Among these factors, bacterial-induced endothelial injury is a significant trigger factor of POVT [3]. However, the pathophysiology of UTI-induced OVT is not established. Anatomically, venous blood of the ureter drains through the ovarian vein, renal vein, and vesical venous plexus before entering inferior vena cava [4]. Therefore, we are considering that the OVT was caused by venous bacterial spread from the ureteral vein to the ovarian vein. However, other possible causal mechanisms can be considered: hemodynamic change of ovarian vein caused by adjacent ureteral inflammation and venous bacterial spread from a renal vein.

As far as we know, there has been only one other case report of UTI-induced OVT. The authors assumed that infection was the cause of the hypercoagulable state, which causes OVT [2]. In addition to a hypercoagulable state, we speculate that venous bacterial spread from the ureteral vein to the ovarian vein could also be the cause of OVT.

CT is the most reliable technique for diagnosing OVT. The image findings include filling defects in a tubular structure with a central round hypoattenuated area and peripheral enhancing rim [5]. In patients with thrombophlebitis, enhancing venous wall thickening and perivenous inflammatory fat infiltration can be identified [6]. MRI is usually not used for early diagnosis. The field of view of ultrasound is often affected by the surrounding organs. Therefore, in most cases, ultrasonography is not appropriate for examining the ovarian vein [6]. In general, it is not difficult to diagnosis OVT by CT scan. However, it is necessary to carefully trace venous anatomy to prevent misinterpretation of OVT to the duplicated ureters in the axial image (***Figure 1B***).

There is only one case report for OVT caused by UTI. However, OVT caused by UTI is not an extraordinary phenomenon. It is a phenomenon that is likely to occur naturally and may have been reported rarely compared to the actual incidence. Therefore, radiologists need to be familiar with that OVT can occur as a complication of UTI.
